# Characteristic Functional Genera (CFG) Mediate Nitrogen Priming Effect in the Microbiome of Saline–Alkaline Farmland

**DOI:** 10.3390/plants14121806

**Published:** 2025-06-12

**Authors:** Yicong Li, Yao Xiao, Wei Zhao, Jiarui Kang, Kejun Yang, Jian Fu

**Affiliations:** 1 College of Agronomy, Heilongjiang Bayi Agricultural University, Daqing 163000, China; lyc0104192001@163.com (Y.L.); 18704641635@163.com (W.Z.); 18345959785@163.com (J.K.);; 2 Key Laboratory of Low-Carbon Green Agriculture in Northeastern China, Ministry of Agriculture and Rural Affairs P. R. China, Daqing 163000, China

**Keywords:** nitrogen fertilizer, saline–alkali land, priming effect, rhizosphere soil, microbial community structure

## Abstract

This study aimed to investigate the impact of nitrogen priming effect on the makeup of the maize rhizosphere microbial community structure in saline–alkali agriculture, focusing on characteristic functional genera. In 2020, three nitrogen levels of 60 kg·ha^−1^ (N1), 180 kg·ha^−1^ (N2), and 300 kg·ha^−1^ (N3), along with a control group, were established in the meadow saline–alkali soil farmland of Daqing in Heilongjiang Province. The maize cultivar was Xianyu 335. Rhizosphere soil was taken for nutritional analysis and high-throughput sequencing of the microbial population. The findings indicated that the bacterial community structure in the N1 and N2 treatment groups was comparable; however, the N3 treatment dramatically altered the community structure (*p* < 0.01). A notable disparity existed between the fungal nitrogen application group and the control group. Screening identified ten genera, including *Lysobacter* and *Coniophora*, as characteristic functional genera, with their habitats and functions dramatically altered during nitrogen priming effect. Nitrogen priming effect enhanced bacterial functionality for nitrogen source augmentation but diminished the capacity for nitrogen transformation, while also altering the nutritional preferences of fungus. Soil nitrogen and organic matter content showed distinct responses to different nitrogen application rates and exhibited significant interactions with the microbial community. The impacts of low, medium, and high nitrogen treatments on microbial and soil indicators varied, suggesting that effective nutrient management necessitates the regulation of microbial community function and accurate nitrogen administration. The research findings hold substantial importance and promotional potential for the sustainable advancement of saline–alkali agriculture.

## 1. Introduction

Soil salinization, a global environmental issue, significantly jeopardizes food security worldwide [1]. Soda saline–alkali soil is characterized by high alkalinity, inadequate water permeability, and suboptimal physical and chemical properties. Saline–alkali conditions will inhibit the growth of crop seedlings and diminish grain output [2]. The elevated pH and salinity levels in saline–alkali soil impede the migration and transformation efficiency of nitrogen, leading to an average nitrogen use efficiency in crops of merely 20% to 25%. This is markedly lower than that in normal soil, resulting in the excessive application of nitrogen fertilizers, which severely impacts agricultural ecology and food security [3].

The priming effect (PE) denotes the alteration in the decomposition rate of original organic matter in soil due to the addition of fresh organic matter or nitrogenous compounds [4]. The priming effect in soil predominantly takes place in the rhizosphere, detrital margins, animal burrows, and other biological voids. The rhizosphere, serving as the primary interface between plant roots and soil, has a limited spatial extent but is significantly influenced by the frequent absorption of nutrients and secretion of substances by roots. This leads to dynamic fluctuations in the distribution of resources, such as carbon and nutrients, making it a focal area for microbial biomass and activity [5]. From the rhizosphere perspective, nutrient competition between roots and microorganisms is a critical element that induces the priming effect. The nutrient mining method frequently induces a favorable priming effect. Nonetheless, when both are significantly constrained by nutrition, a negative priming effect may also manifest [6]. Investigating the determinants and ecological mechanisms of PE is crucial for sustaining long-term soil carbon sequestration and preserving ecosystem stability [7]. Over the past twenty years, PE has emerged as a central focus of research within soil ecology.

In agricultural ecosystems, exogenous nitrogen serves as a significant catalyst for soil nitrogen priming, and its impact on the soil nitrogen priming effect has emerged as a focal point of research [8]. A multitude of studies have demonstrated that the utilization of inorganic nitrogen fertilizer enhances the natural nitrogen content in soil. Jenkinson et al. discovered that the release of unlabeled inorganic nitrogen from soil escalated following fertilizer [9]. Nonetheless, there exists a perspective suggesting that this phenomenon may stem from the substitution of fertilizer nitrogen and intrinsic nitrogen, implying that the introduction of external nitrogen may not genuinely enhance the availability of nitrogen in the soil. The research conducted by He indicated that the application of nitrogen fertilizer did not expedite the decomposition of soil organic nitrogen. It markedly enhanced the microbial biological fixation of mineral nitrogen in the soil [10], thereby sparking debate regarding the underlying nature of the positive priming effect associated with nitrogen fertilizer. In conclusion, while the introduction of exogenous inorganic nitrogen typically enhances soil nitrogen turnover, notable variations exist in the priming effect, and the interconversion characteristics of soil nitrogen mineralization and fixation are regarded as critical determinants influencing this effect.

Current research predominantly examines neutral soil, with limited studies addressing the impact of nitrogen priming effect in saline–alkali agricultural land. There is a lack of extensive research on the alterations in microbial community structure and function within the rhizosphere of maize in saline–alkali agriculture, and the examination of microbial function is superficial and incomplete.

This research presents a novel examination of saline–alkali agriculture, providing a detailed analysis of the microbial community and soil conditions at varying nitrogen levels. We integrated LDA effect analysis with a random forest model to identify the genus and thoroughly investigate its niche, functional transformation, nitrogen source utilization preference, and further characteristics. Through comparison with prior studies, we established its distinct importance and value in microbial ecology and nitrogen management of crops in saline–alkali environments.

According to the principle of niche and resource competition, it is hypothesized that nitrogen gradient input will variably influence the composition of the rhizosphere microbial population. The high nitrogen treatment markedly differs from the low and medium nitrogen groups due to heightened resource competition and disruption of community equilibrium. According to the notion of microbial functional adaptability, the response of typical characteristic functional genera to nitrogen levels is anticipated to be functionally distinctive. Nitrogen fixation and organic matter breakdown occur at low nitrogen concentrations, while nitrogen assimilation processes are intensified at elevated nitrogen levels. The nitrogen priming effect, in conjunction with soil fertility formation and microbial-mediated processes, altered soil carbon and nitrogen transformations through microbial functions. Elevated nitrogen levels inhibited soil carbon and nitrogen fixation capabilities, thereby diminishing the stability of soil organic matter.

## 2. Materials and Methods

### 2.1. Test Sites and Varieties

The sampling site is situated in Daqing City, Heilongjiang Province, China (46°37′ N, 125°11′ E), at an elevation of 152 m (a.s.l.), characterized by a flat alluvial plain with a slope of less than 1°, exhibiting no significant incline. The test field is encircled by adjacent corn cultivation zones devoid of natural vegetation cover. The test area is classified as Dwa (cold temperate continental monsoon climate) according to the Köppen climatic classification, featuring an average annual temperature of 4.5 °C, annual precipitation of 427.5 mm, annual evaporation of 1635 mm, and a frost-free duration of 143 days. The minimum temperature is −39.2 °C, while the maximum temperature is 39.8 °C. The test site comprises Meadow Solonchak soil (Table S1). The maize variety evaluated is Xianyu 335 (*Zea mays* L.).

### 2.2. Experimental Design

The experiment commenced on 16 May 2020, utilizing a randomized block design. Each treatment was replicated three times, establishing three separate experimental plots. Each independent experimental plot was subdivided into four distinct experimental plots. Each experimental plot encompassed an area of 56 m^2^, comprising a total of 8 ridges measuring 0.7 m by 10 m. Maize seeds were planted at a density of 82,500 plants per hectare with a row spacing of 0.7 m.

The nitrogen application rates were 60 kg·ha^−1^ (N1), 180 kg·ha^−1^ (N2), and 300 kg·ha^−1^ (N3), respectively. The control group did not utilize nitrogen fertilizer (Table 1). The treatment employed a high-yield fertilizer regimen, utilizing a quarter of the nitrogen application rate of the test treatment, alongside a total application of phosphorus and potassium fertilizers (90 kg P2O5 ha^−1^ and 120 kg K2O ha^−1^) as the foundation fertilizer. At the seven-leaf stage, 50% of the experimental treatment was administered with nitrogen fertilizer, and at the tasseling stage, 25% of the experimental treatment was supplied with nitrogen fertilizer (46% N).

After hand thinning, one plant was retained at each planting location throughout the two-leaf stage. After fertilization at the silking stage (VT), standardized irrigation (20 mm) was performed after fertilization to ensure that nitrogen penetrated into the rhizosphere and then sampled. Supplementary management techniques, including pest and weed control, were executed in compliance with local agronomic requirements unless stated otherwise.

### 2.3. Rhizosphere Soil Sampling

In 2020, five-point sampling was conducted for each experimental plot during the tasseling (VT) and maize maturation phase (R6). Maize plants were excavated from the soil, and larger soil particles attached to the roots were meticulously removed. Subsequently, fine brushes were used to collect the rhizosphere soil adhering to the fine roots into sterile, self-sealing plastic bags. A portion of the soil samples was stored in a −80 °C freezer for high-throughput sequencing analysis of soil bacterial and fungal communities. The leftover soil samples were desiccated in air.

### 2.4. Determination of Soil Nutrient Content

Soil organic matter (OM) was quantified using the K_2_Cr_2_O_7_H_2_SO_4_ digestion method [11]. Total nitrogen (TN) was quantified utilizing the Kjeldahl-N technique. Available nitrogen (AN) was extracted using 1 M KCl and analyzed via the cadmium reduction method [12].

### 2.5. High-Throughput Sequencing Analysis of Bacterial and Fungal Communities in Rhizosphere Soil

Genomic DNA from soil was isolated using a Power Soil DNA Isolation Kit (MoBio Laboratories Inc., Carlsbad, CA, USA) following the manufacturer’s instructions. The concentration and purity (A260/A280 ratio) of the DNA samples were evaluated using a NanoDrop 2000 spectrophotometer (Thermo Scientific, Waltham, MA, USA). Pyrosequencing analyses of the 16S rRNA gene and ITS region were performed to determine the diversity and composition of bacterial and fungal communities, respectively. The V3–V4 region of the bacterial 16S rRNA gene was amplified via PCR with primers 338F (5′-ACTCCTACGGGAGGCAGCAG-3′) and 806R (5′-GGACTACHVGGGTWTCTAAT-3′), while the ITS1 segment of the fungal ITS was amplified using primers ITS1-F (5′-CTTGGTCATTTAGAGGAAGTAA-3′) and ITS2R (5′-TGCGTTCTTCATCGATGC-3′). The PCR products were purified utilizing a PCR Purification Kit (Axygen Bio, Union City, CA, USA) and quantified with PicoGreen^®^ dsDNA reagent (Promega, Madison, WI, USA). The purified amplicons were then merged in equimolar amounts into a single aliquot and employed for library construction using the NEB Next^®^ Ultra^TM^ DNA Library Prep Kit for Illumina (New England Biolabs, Ipswich, UK). All library preparation was performed on the Illumina MiSeq platform.

The sequences retained for each sample were examined with the UPARSE methodology, incorporating USEARCH and Perl scripts to generate a table of operational taxonomic units (OTUs) and to select representative sequences. The standard operating method of MOTHUR (version 1.25.1, Ann Arbor, MI, USA) was employed for the subsequent analysis of pyrosequencing data. To correct the sampling effort, a minimum of 11,616 and 44,294 sequences were randomly selected from each sample for the subsequent investigation of bacterial and fungus populations.

### 2.6. Data Processing and Analysis

R 4.4.1 was employed to examine shared and exclusive species data, community composition, and the species abundance matrix for OTU categorization. usearch-alpha_div (V10) in R was used to calculate the Chao1 and Shannon alpha diversity indices, and PCA analysis was carried out using prcomp. Linear Discriminant Analysis Effect Size (LEfSe) identified significant microbial groupings, yielding LDA bar graphs with LDA values above 3. randomForest was employed to construct predictive models, assess primary species, and tailor validation set results, emphasizing “Mean Decrease Accuracy” and “Mean Decrease Gini”. FAPROTAX and FUNGuild were employed to forecast bacterial and fungal functionalities. DCA was performed utilizing R software 4.4.1 to amalgamate soil nutrient and aggregate data, with the RDA model predicated on gradient values. Analysis and visualization were conducted to generate heatmaps and correlate community structure with environmental variables. Path analysis employs specialized abilities to delineate variable relationships, produce a route diagram, and formulate a multiple linear regression model to elucidate both direct and indirect connections. The model is further refined according to its quality of fit, statistical significance, and expert knowledge. A more straightforward model that aligns professional expertise with data attributes was developed following numerous attempts. Excel structures data, SPSS 27.0 and Excel 2010 generate tables, whilst Origin 2021 produces scientific charts.

## 3. Results

### 3.1. Effect of Nitrogen Priming on Microbial Dominant Flora Community

**Figure 1:**
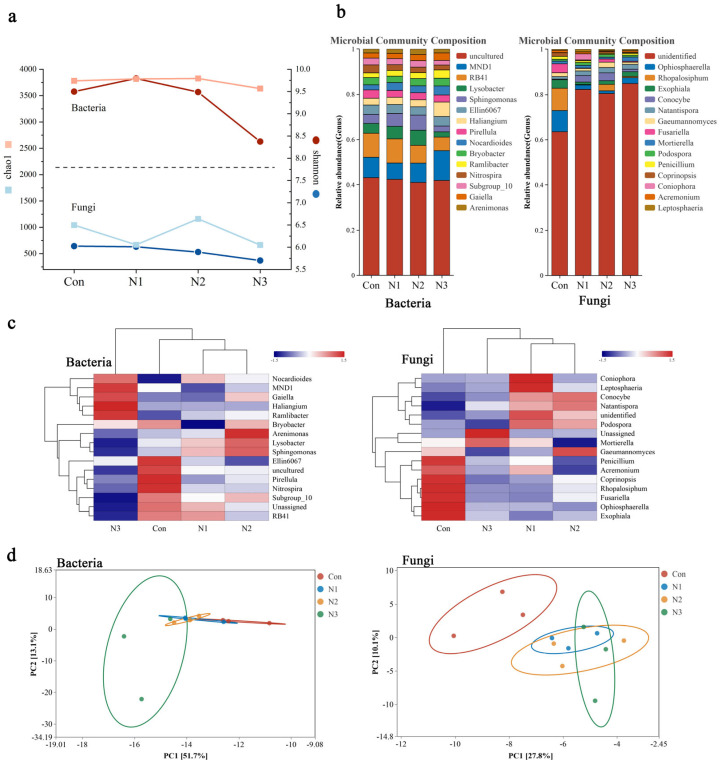
(**a**): Line chart depicting the alpha diversity indices (Chao1 and Shannon) of maize rhizosphere soil microorganisms at varying nitrogen levels. (**b**): The relative abundance map of the predominant microbial species (top 15 in abundance) in the rhizosphere soil of maize at varying nitrogen levels. (**c**): Cluster analysis of the abundance of dominating microbial species in maize rhizosphere soil across varying nitrogen levels. (**d**): Principal component analysis of maize rhizosphere soil microorganisms under varying nitrogen levels, based on Euclidean metrics. Control (Con), N1 (60 kg·ha^−1^), N2 (180 kg·ha^−1^) and N3 (300 kg·ha^−1^).

Initially, regarding α diversity (Figure 1a), the Shannon index of bacteria peaked in the N1 treatment and then declined with the escalation of nitrogen application rates, particularly in the N3 treatment. The Chao1 index of microorganisms exhibited no significant differences among the treatment groups. The fungal community’s Shannon index decreases with increasing nitrogen application rates; the Chao1 index under N2 treatment exhibits some advantages, yet overall diversity is significantly suppressed by higher nitrogen application rates.

The bacterial-dominating flora community (Figure 1b) exhibited a considerable increase in *Sphingomonas* abundance by 43.09% and 68.15% after N1 and N2 treatments, respectively, compared to the control. Nonetheless, its prevalence was markedly diminished by 35.22% with N3 therapy. The proliferation of *Ramlibacter* rose by 24.16%, 26.65%, and 78.40% under treatments N1, N2, and N3, respectively. The prevalence of *Nitrospira* diminished by 28.66%, 31.40%, and 38.97% with treatments N1, N2, and N3, respectively. The abundance of certain dominating genera within the fungal community considerably diminished with increased nitrogen fertilizer application. The abundance of *Penicillium* diminished by 36.72% to 66.41%, *Ophiosphaerella* decreased by 72.22% to 87.50%, *Rhopalosiphum* declined by 71.40% to 98.07%, and *Exophiala* reduced by 44.44% to 70.37%. The proliferation of *Conocybe* markedly escalated following nitrogen administration, with an increase ranging from 319.23% to 1230.77%.

In the bacterial cluster analysis (Figure 1c), the N1 and N2 treatment groups exhibited clear aggregation, whereas the N3 treatment significantly influenced the community structure of the dominant bacterial flora, particularly in comparison to the control, where the community structure underwent notable alterations. The outcomes of the principal component analysis (Figure 1d) and species abundance cluster analysis corroborated one another. The fungal cluster analysis revealed that the N1 and N2 treatment groups were initially densely clustered, exhibiting a high degree of similarity. The two polymers were grouped with the N3 treatment cohort, suggesting that the three nitrogen treatment groups exhibited certain commonalities collectively. Moreover, the three nitrogen application groups exhibited substantial differences from the control group, indicating that the predominant fungal community was markedly responsive to nitrogen fertilizer. This conclusion was further corroborated by PCA analysis and cluster analysis of species abundance.

### 3.2. Screening of Characteristic Functional Bacteria Under Nitrogen Excitation Effect

**Figure 2:**
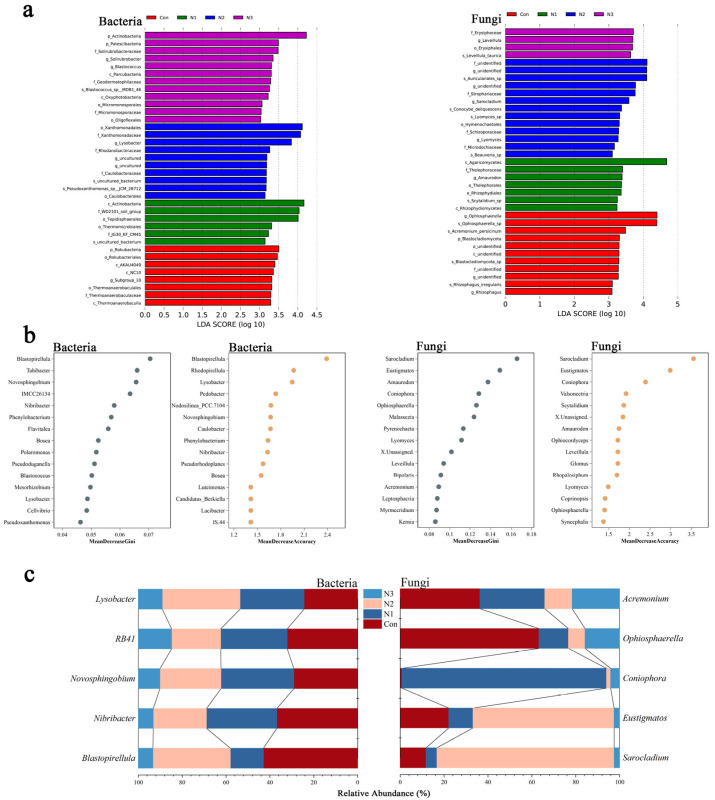
(**a**): The distribution histogram of microbial LDA values (LDA values exceeding 3) in maize rhizosphere soil across varying nitrogen levels. (**b**): Random forest study of microbial species in maize rhizosphere soil across varying nitrogen levels. Random forest was employed to identify the top 15 genera from a multitude of microbiological genera. (**c**): Characteristics of maize rhizosphere soil at varying nitrogen levels; histogram of bacterial relative abundance. Control (Con), N1 (60 kg·ha^−1^), N2 (180 kg·ha^−1^) and N3 (300 kg·ha^−1^).

Utilizing LDA effect size analysis and a random forest model, representative and specific genera in the rhizosphere soil of maize cultivated in saline–alkali farmland were effectively identified and accurately classified based on their significant abundance (Figure 2), thereby designating them as characteristic functional genera.

In terms of functional bacterial genera, the abundance of *Blastopirellula* and *Nibribacter* under N1 and N3 conditions was lower than that of Con, with the decline intensifying as nitrogen levels increased, suggesting that nitrogen impeded their growth. The abundance of *Novosphingobium* rose by 14.4% under N1 conditions and declined under N2 and N3 conditions, suggesting that moderate nitrogen fosters growth, whereas excessive nitrogen suppresses it. The decline in *RB41* persisted as nitrogen levels rose, suggesting that the inhibitory effect of increased nitrogen on its growth was intensified. The proliferation of *Lysobacter* augmented under N1 and N2 settings, whereas it diminished under N3 conditions, suggesting that a specific nitrogen range facilitated growth, whilst exceeding that range resulted in inhibition.

The abundance of the fungal genera *Sarocladium* and *Eustigmatos* dramatically increased under N2 conditions, rising by 585.5% and 190.6%, respectively, demonstrating that N2 treatment had a distinct stimulatory effect on their growth. The proliferation of *Coniophora* surged by 12,207.7% with N1 treatment, signifying that N1 treatment significantly enhanced its growth. *Ophiosphaerella* and *Acremonium* exhibited strong sensitivity to nitrogen, with their abundance declining under varying amounts of nitrogen fertilizer.

### 3.3. Effect of Nitrogen Priming on Microbial Function Prediction

**Figure 3:**
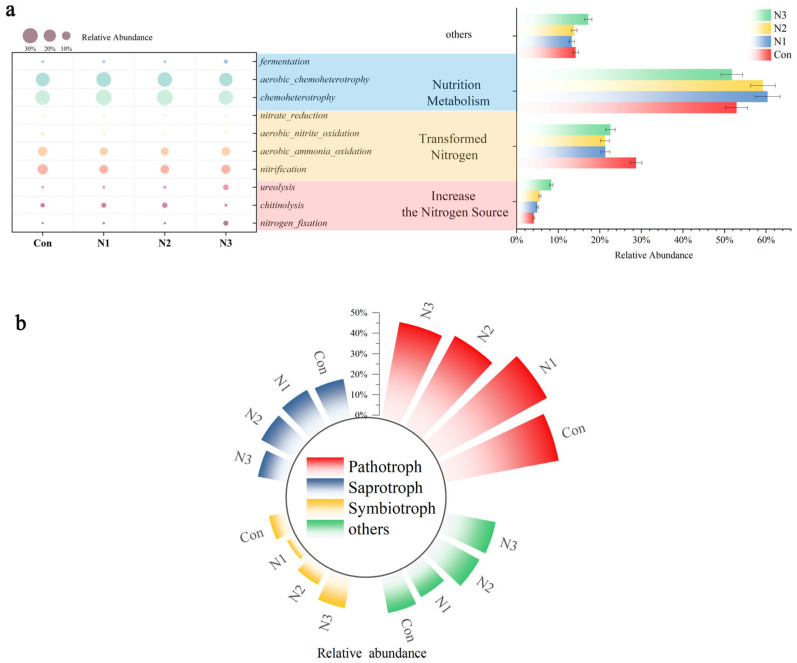
(**a**): Predicted functional profiles of bacterial community in maize rhizosphere soil under different nitrogen application rates. (**b**): Forecasting the functional capabilities of fungal communities in maize rhizosphere soil at different nitrogen concentrations (pathotroph, saprotroph, and symbiotroph). Control (Con), N1 (60 kg·ha^−1^), N2 (180 kg·ha^−1^) and N3 (300 kg·ha^−1^).

In the context of bacterial function prediction (Figure 3a), the treatment conditions N1, N2, and N3 demonstrated a considerable enhancement in function due to the increased nitrogen source, with improvement rates of 20.48%, 35.42%, and 101.45%, respectively, compared to the control group. Nitrogen fixation increased by 21.05%, 22.81%, and 473.68% under treatments N1, N2, and N3, respectively. The chitin decomposition function increased by 20.22% and 35.21% under N1 and N2 treatments, respectively, but reduced by 62.17% after N3 treatment. The enhancement of urea decomposition function under treatments N1, N2, and N3 was 19.78%, 43.96%, and 347.25%, respectively. Contrary to the increased nitrogen source, the transformation of nitrogen functions exhibited a decline under N1, N2, and N3 treatments, with reductions of 25.69%, 26.00%, and 21.44%, respectively. N1 and N2 treatments enhanced nutritional metabolism ability by 14.12% and 12.02%, respectively, while N3 treatment resulted in a decrease of 2.12%. The alterations in fermentation function under varying nitrogen treatments exhibited a distinct pattern. Under N1 treatment, the fermentation function rose by 16.47%; it reduced by 1.18% under N2 therapy. Nonetheless, it rose by 138.82% with N3 therapy.

In fungal function prediction (Figure 3b), phototroph increased by 9.13% with N1 treatment, but it declined by 10.21% and 15.42% under N2 and N3 treatments, respectively. The saprotroph increased by 5.23% with N1 treatment, declined by 2.57% under N2 treatment, and exhibited a more pronounced decline of 27.26% during N3 treatment. The evolving tendency of symbiotrophs is increasingly distinctive. It diminished by 51.31% following N1 therapy, but it augmented by 5.96% and 83.00% under N2 and N3 treatments, respectively.

### 3.4. Effects of Nitrogen Priming on Soil Nitrogen and Organic Matter

**Figure 4:**
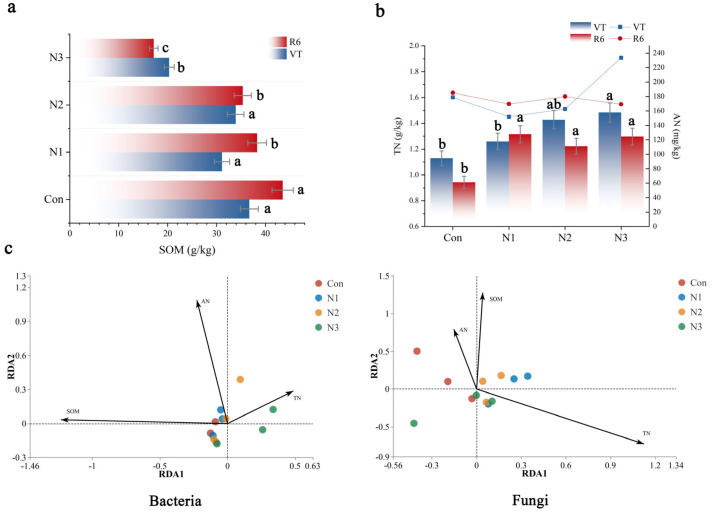
(**a**): The concentrations of alkali-hydrolyzable nitrogen and total nitrogen in maize rhizosphere soil at varying nitrogen levels. (**b**): Organic matter concentration in maize rhizosphere soil at varying nitrogen levels. Different lowercase letters indicated that there were significant differences between different treatments (*p* < 0.05). (**c**): RDA study of microbial and soil nitrogen, as well as organic matter, in maize rhizosphere soil at varying nitrogen levels. Control (Con), N1 (60 kg·ha^−1^), N2 (180 kg·ha^−1^) and N3 (300 kg·ha^−1^).

In the N1 and N2 treatments, the soil organic matter at the mature stage (R6) exceeded that at the tasseling stage (VT) (Figure 4a); conversely, in the N3 treatment, the soil organic matter at the mature stage was inferior to that at the tasseling stage. Furthermore, at the tasseling stage, the soil organic matter content in the N3 treatment was 34.72% to 40.09% lower than that of other low nitrogen treatments; at the ripening stage, the N3 treatment exhibited a more significant decline compared to other low nitrogen treatments, ranging from 51.46% to 55.20%.

The trend in total nitrogen content in the soil mirrored that of soil organic matter content (Figure 4b). Regarding soil total nitrogen content, the mature period of the N1 treatment exceeded that of the tasseling period; conversely, under N2 and N3 treatments, the soil total nitrogen content at full maturity was inferior to that at the tasseling stage. The concentration of alkali-hydrolyzable nitrogen in the N1 and N2 treatments was markedly greater than that observed during the tasseling stage. Conversely, it exhibited an inverse pattern with N3 treatment, with the maturity time being shorter than the tasseling period. During the tasseling stage, the alkali-hydrolyzable nitrogen content in the soil rose to the elevated nitrogen input levels. At the mature stage, there was no notable change in soil alkali-hydrolyzable nitrogen content across the nitrogen treatments.

The RDA analysis of bacterial and fungal communities (Figure 4c) indicated that N1 and N2 treatments had a beneficial impact on soil organic matter and alkali-hydrolyzed nitrogen. The acute angle between alkali-hydrolyzable nitrogen and the organic matter vector indicates a synergistic interaction between the two in influencing bacterial and fungal communities. In the RDA diagram of bacteria, the organic matter vector is markedly longer than other components, and alterations in its content will substantially affect the distribution of bacterial communities. The total nitrogen (TN) vector in the fungal RDA map is extensive, signifying that total nitrogen significantly influences the organization of the fungal community.

### 3.5. Path Analysis of Different Nitrogen Levels Under Nitrogen Excitation Effect

**Figure 5:**
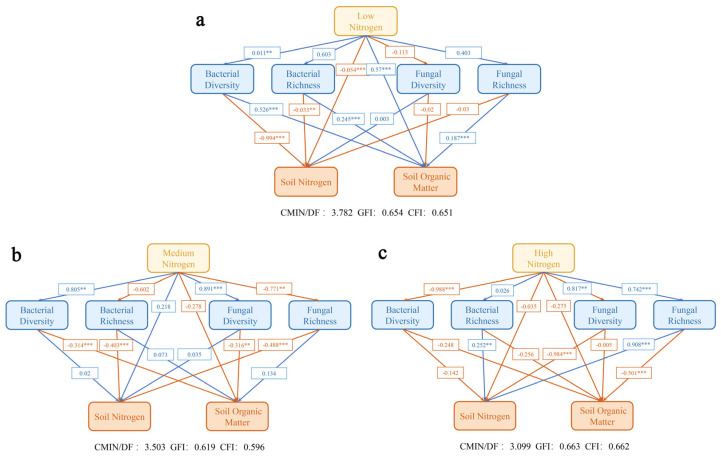
The path analysis of soil microorganisms, alkali-hydrolyzed nitrogen, and soil organic matter at low nitrogen (**a**), medium nitrogen (**b**), and high nitrogen (**c**) levels. The blue line denotes a positive correlation, while the orange line indicates a negative correlation, represented as a standardized coefficient on the arrow. CMIN/DF represents the chi-square degree of freedom ratio, GFI denotes the goodness of fit index, CFI signifies the comparative fitting index, and the overall adequacy of the equation is assessed comprehensively. ** and *** represent the significance level of 5% and 1% respectively. Low nitrogen (60 kg·ha^−1^), medium nitrogen (180 kg·ha^−1^) and high nitrogen (300 kg·ha^−1^).

At low nitrogen levels (Figure 5a), the direct effect of low nitrogen treatment significantly enhanced bacterial diversity (0.011, *p* < 0.01) and exerted a significant negative effect on soil nitrogen (−0.054, *p* < 0.001) while positively influencing soil organic matter (0.57, *p* < 0.001). Given that the path coefficient of low nitrogen treatment on certain microbiological indicators was not substantial, the indirect influence was challenging to elucidate. Regarding the connection of microorganisms with soil indicators, bacterial diversity (−0.994, *p* < 0.001) and bacterial richness (−0.033, *p* < 0.01) exhibited substantial negative direct effects on soil nitrogen. Bacterial diversity (0.526, *p* < 0.001), bacterial richness (0.245, *p* < 0.001), and fungal richness (0.187, *p* < 0.001) had significant beneficial direct effects on soil organic matter. The low nitrogen treatment had a favorable effect on bacterial diversity, a negative effect on soil nitrogen, and a positive effect on soil organic matter.

At a medium nitrogen level (Figure 5b), medium nitrogen exerted a substantial positive direct influence on bacterial diversity (0.805, *p* < 0.01) and fungal diversity (0.891, *p* < 0.001), while having a significant negative direct effect on fungal richness (−0.771, *p* < 0.01). Bacterial diversity (−0.314, *p* < 0.001) and fungal diversity (−0.316, *p* < 0.01) had a substantial negative direct effect on soil organic matter, while bacterial richness (−0.403, *p* < 0.001) and fungal richness (−0.488, *p* < 0.001) negatively impacted soil nitrogen. Medium nitrogen may indirectly influence soil organic matter through its impact on microbial diversity, or alter soil nitrogen via fungal richness. The overall impact of medium nitrogen on bacterial and fungal diversity was positive, whereas it negatively affected fungal richness, soil organic matter, and soil nitrogen, resulting from both direct and indirect effects.

At elevated nitrogen concentrations (Figure 5c), high nitrogen exhibited a substantial negative direct impact on bacterial diversity (−0.988, *p* < 0.001), while demonstrating a considerable positive direct influence on fungal diversity (0.817, *p* < 0.01) and richness (0.742, *p* < 0.001). Bacterial richness exerted a significant positive direct influence on soil nitrogen (0.252, *p* < 0.01). Fungal richness exerted negative direct impacts on soil organic matter (−0.501, *p* < 0.001) and positive direct effects on soil nitrogen (0.908, *p* < 0.001), respectively. The diversity of fungi exerted a significant negative direct influence on soil nitrogen (−0.984, *p* < 0.001). The indirect effect involves the detrimental impact of elevated nitrogen levels on bacterial diversity and the beneficial influence on fungal diversity and richness, which subsequently influenced soil attributes via the interaction between microorganisms and soil indicators. The overall impact of elevated nitrogen on bacterial diversity was detrimental, but its influence on fungal diversity and richness was beneficial. Additionally, the total effect on soil nitrogen was favorable, although the impact on soil organic matter was negative.

## 4. Discussion

### 4.1. Effects of Nitrogen Priming on Microbial Community Structure and Dominant Flora

Various nitrogen levels markedly altered the microbial community structure in the maize rhizosphere of saline–alkali cropland (Figure 1). Elevated nitrogen (N3) intake significantly disrupted the bacterial community structure, while nitrogen fertilizer application was instrumental in influencing the fungus community. This structural difference significantly influences microbial niche distribution and species coexistence patterns from an ecological perspective. Microorganisms can persist in low and medium nitrogen circumstances due to analogous resource consumption traits, consistent environmental selection pressure, and modest niche overlap. Elevated nitrogen treatment results in heightened resource competition and significant alterations in environmental parameters, prompting microbial niche diversification and recombination [13]. For instance, certain oligotrophic microbes such as *Sphingomonas* diminish in high nitrogen environments, but the niche of eutrophic microorganisms proliferates, altering community composition and diversity patterns [14].

The predominant microorganisms had varying sensitivities to nitrogen concentrations. *Sphingomonas* thrives in medium and low nitrogen environments, whereas high nitrogen levels restrict its growth, potentially due to the regulation of nitrogen metabolism and disruption of osmotic pressure balance [15]. The *Ramlibacter* genus thrived under all nitrogen treatments, demonstrating significant adaptability and a favorable response mechanism to nitrogen enhancement, and predominated in nitrogen-rich soil due to its effective nitrogen uptake system [16]. The persistent decline in *Nitrospira* indicates that the elevated nitrogen environment impedes its nitrification capability and disrupts the soil nitrogen transformation process [17]. The prevalence of *Penicillium, Ophiosphaerella, Rhopalosiphum*, and *Exophiala* among the predominant fungal communities usually diminished, whereas the genus *Conocybe* had a large rise, suggesting that nitrogen administration altered the competitive dynamics of the fungal communities. *Conocybe* may be stimulated by nitrogen to improve colonization and reproduction in the rhizosphere [18], and the alterations in dominant flora are intricately linked to soil nitrogen dynamics and saline–alkali conditions, collectively influencing the rhizosphere microecology.

### 4.2. Characteristics and Functions of Characteristic Functional Genera Under Nitrogen Priming Effect

The soil microbial community with exogenous nitrogen addition and the maize rhizosphere soil microbial community exhibit distinct traits and compositions. Through their interactions, they guide and transform into a novel microbial community. Consequently, we propose a specialized set that highlights the importance of these genera in terms of abundance, as well as their representativeness and specificity within a particular environment (saline–alkali agricultural maize rhizosphere soil), thereby establishing a new specific set: characteristic functional genera (Figure 2).

The nitrogen priming effect substantially altered the ecological and functional roles of the characteristic functional genera. Bacterial taxa like *Blastopirellula* are integral to the nitrogen cycle [19]. Low nitrogen levels may enhance nitrogen fixation and augment soil nitrogen; high nitrogen levels regulate nitrogen metabolism balance, prevent excessive nitrogen accumulation or transformation imbalances, optimize nitrogen utilization pathways through gene expression regulation and alterations in enzyme activity, and influence nitrogen acquisition and the community structure of surrounding microorganisms [20]. Fungal characteristic functional genera, such as *Sarocladium*, exhibit dynamic alterations in organic matter breakdown and plant hormone control. Nitrogen alterations influence the secretion of extracellular enzyme activity and the expression of hormone synthesis genes [21], subsequently regulating the cadence of soil nutrient release and the growth and development of plant roots. The connection between the mycelium network and the root system, together with other microorganisms, initiates a chain reaction in the nutrient cycle and energy flow of the ecosystem, serving as a crucial node for maintaining the stability of rhizosphere ecology.

The characteristic functional genera exhibit varying preferences for nitrogen source consumption and exhibit precise metabolic regulation. *RB41* exhibits a pronounced affinity for ammonium nitrogen, with the expression of ammonium transporter gene family members being stimulated in low ammonium conditions, facilitating the active uptake of ammonium ions [22]. Conversely, when nitrogen levels rise, the expression of this gene is suppressed through negative feedback, leading to the up-regulation of nitrate reductase gene transcription, thereby transitioning to a nitrate nitrogen utilization mode to enhance nitrogen source utilization efficiency and maintain intracellular nitrogen metabolic homeostasis. The metabolic pathway is regulated according to the stoichiometric ratio of the nitrogen source and its requirements, which is a crucial factor influencing the dynamic changes in microbial community structure under nitrogen excitation [23]. The proliferation of *Eustigmatos* markedly escalated following N2 treatment, signifying that this therapy exerted a distinct stimulatory effect on its growth. In both low and high nitrogen settings, its abundance remains comparatively low. In an appropriate nitrogen environment (e.g., N2 treatment), it can influence the soil microenvironment via its metabolic activities, including nitrogen absorption and transformation, regulation of nitrogen forms and content in the soil, and provision of optimal nitrogen nutrition for adjacent microorganisms and plants [24].

The nitrogen priming effect process modifies the metabolism of characteristic functional genera, hence altering the soil microenvironment and being regulated by its feedback mechanisms. Using *Nibribacter* as an example, its metabolic acid production lowered the pH of rhizosphere soil, altered the kinetics of soil mineral dissolution and nutrient release, and influenced the transformation and availability of nitrogen forms. The reduction in soil pH subsequently influences its cell membrane potential and enzymatic activity, necessitating the regulation of metabolic pathways to respond to environmental alterations [25]. The fluctuation of redox potential, induced by the metabolism of characteristic functional genera, influences the circulation of valence elements and microbial respiration in soil, creating a complex feedback loop that significantly impacts the ecological function and structure of microbial communities, thereby dictating the trajectory of soil ecological processes under nitrogen excitation [26]. The metabolic activities of characteristic functional genera engage with the soil environment, akin to the microbial secretion of extracellular enzymes that decompose both exogenous organic matter and soil organic matter, as described in the co-metabolism theory [27]. The metabolites of characteristic functional genera alter the soil environment, therefore influencing the usage and transformation of soil organic matter and nutrients by both themselves and other microbes.

In the soil ecosystem, *Ophiosphaerella* and *Acremonium* exhibited sensitivity to variations in nitrogen concentrations. The application of nitrogen fertilizer diminished their abundance, with a more pronounced reduction correlating with increased nitrogen content. The nitrogen priming effect presumably alters the competitive dynamics among soil microorganisms, enhancing their adaptability to nitrogen-rich environments. The genus *Ophiosphaerella* is notably pathogenic to significant crops [28]. Infection by this genus in plants results in symptoms like premature wilting of leaves and ears, root rot, and maturation abnormalities, significantly jeopardizing crop growth and output. *Acremonium* possesses the capability to decompose pesticides in soil, hence significantly diminishing pesticide residues. It is crucial for soil environmental restoration and the maintenance of ecological balance, significantly enhancing soil quality and fostering sustainable agricultural development [29].

Characteristic functional genera possess the capacity to serve as crucial biological markers of soil quality, nitrogen use efficiency, and ecosystem health on saline–alkali agricultural land. Its prevalence and functional alterations closely mirror soil nitrogen transformation, organic matter breakdown, and microbial community equilibrium. For instance, the abundance of *Novosphingobium* was significantly correlated with the dynamics of soil nitrogen mineralization and fixation, and its reduction under N3 treatment may signify substantial alterations in soil nitrogen availability and microbial-driven nitrogen turnover efficiency [30]; the functional stability of *Sarocladium* in regulating plant hormones and engaging in nutrient cycling can delineate the integrity of ecosystem services [31]. Long-term monitoring of its dynamic changes offers a sensitive and reliable biological foundation for early detection of soil degradation, optimization of nitrogen management strategies, and assessment of ecological restoration efficacy, thereby significantly aiding scientific decision-making for sustainable agricultural development in saline–alkali regions.

### 4.3. Functional Analysis of Bacteria and Fungi Under Nitrogen Priming Effect

The predictive outcomes of bacterial function indicated its fundamental involvement in soil ecology (Figure 3a). The boost of nitrogen source acquisition and transformation functions, such as nitrogen fixation and urea decomposition, expedited the replenishment of soil inorganic nitrogen and stimulated the increase of nitrogen cycle flow. Functional alterations, such as chitin breakdown, modify the mineralization dynamics of soil organic matter and the nitrogen release pathway, hence influencing the trajectory of soil fertility evolution. It is intricately linked to the characteristic functional genera, such as *Lysobacter*, which can produce various hydrolases to facilitate the degradation of organic materials when nitrogen cycle functionality is augmented. Nitrogen priming effect influences the transcription and translation of genes associated with relevant enzymes [32,33]. In conditions of low nitrogen, it increases the expression of the chitinase gene and decomposes nitrogen-containing organic materials in the soil to release ammonium nitrogen [34]. This procedure adheres to the nutrient mining idea [35]. Microorganisms utilize carbon synthesis and the secretion of extracellular enzymes to degrade specific nitrogenous organic matter, thereby acquiring nutrients and facilitating the carbon and nitrogen cycles, which encourage the release of carbon from soil organic matter. Excess nitrogen impedes this process and leads to the optimization of the nitrogen absorption pathway. By upregulating the ammonia transporter gene to effectively receive and utilize exogenous nitrogen, intracellular nitrogen metabolism flux is enhanced, sustaining growth and competitive advantages, while thoroughly integrating the soil nitrogen cycle and energy metabolism network. Consequently, its nitrogen-fixing capability can serve as a focal point for the advancement of biofertilizers in the administration of nitrogen in saline–alkali soils.

The dynamic alterations in fungal nutritional kinds significantly influence the multi-level environment (Figure 3b). The pathotroph type augmented during N1 treatment and diminished with N2 and N3 treatments, indicating the fungi’s weak pathogenicity or probable symbiotic inclination towards plants in low nitrogen conditions, but high nitrogen mitigated its detrimental effects and promoted plant health preservation. The trend in saprophytic nutrient types indicated that nitrogen administration enhanced organic matter breakdown in the first phase, whereas excessive nitrogen subsequently inhibited this process, hence impacting the dynamic equilibrium of the soil carbon pool and the continuity of nutrient release. The distinctive alteration in symbiotic nutrition type indicates the formation of a close symbiotic association with plant roots in medium to high nitrogen environments, hence augmenting plant nutrient absorption and stress resilience. The characteristic functional genera *Coniophora* has mycelial growth that is intricately associated with soil particles. Nitrogen fluctuations alter the activity of essential enzymes in the nitrogen metabolism pathway, such as nitrate reductase, hence impacting nitrogen uptake and conversion efficiency [36].

In comparison to prior research on saline–alkali land or various agricultural systems, the alterations in microbial communities seen in this experiment exhibited both similarities and distinct features. Nitrogen application typically influences community structure and function [37,38], resulting in the replacement of dominant bacteria and the response of functional bacteria across various systems, thereby illustrating the fundamental ecological feedback mechanism of soil microorganisms to nitrogen input. This study possesses distinctive attributes at the differential level, attributable to the specific soil chemical properties of saline–alkali farmland, the characteristics of maize crop root exudates, the variability of microbial communities, the types of sensitive bacteria, and the trajectory of functional transformation. Bacillus exhibits a vigorous reaction in a saline–alkali environment [39], resulting from the synergistic influence of saline–alkali stress and nitrogen, which alters the survival threshold and metabolic preferences of microorganisms. This study significantly contributes to the microbial ecology theory of saline–alkali land, offering precise guidance for targeted crop nitrogen management practices and accumulating essential data and concepts for cross-ecosystem microbial research.

### 4.4. Complex Correlation Between Soil Fertility and Nitrogen and Analysis of Nitrogen Priming Effect

Soil organic matter serves as a crucial indicator of soil fertility (Figure 4a). In a low-nitrogen environment, the soil microbial population remains stable and extremely active, effectively decomposing organic matter and facilitating its accumulation. Excessive nitrogen input disrupted the soil ecological equilibrium and suppressed the proliferation of some microbial groups vital for organic matter decomposition, leading to expedited organic matter breakdown and postponed synthesis [40]. The trend in total nitrogen content in the soil mirrored that of soil organic matter, suggesting a close correlation between the two (Figure 4b). The N1 treatment consistently correlated with alterations in soil organic matter, further validating that the soil was more favorable for nitrogen accumulation and retention under low nitrogen and ecologically stable conditions. Nonetheless, elevated nitrogen levels and substantial one-time inputs significantly exceed the soil’s self-regulatory capacity. The absorption and use efficiency of nitrogen by crops and soil sequestration cannot match the rapid increase in nitrogen input, resulting in significant amounts of nitrogen being underutilized. The substantial nitrogen loss and crop absorption until maturity lead to a marked decrease in the total nitrogen concentration of the soil. The alkali-hydrolyzable nitrogen content of soil indicates the nitrogen supply capacity of the soil for crops. During the tasseling stage, the content of alkali-hydrolyzable nitrogen in the soil rose in correlation with the augmentation of the nitrogen supply. In the mature stage, the initial supply of soil nitrogen varied, although the concentration of alkali-hydrolyzable nitrogen assimilated by crops became relatively uniform via a sequence of transformations and utilizations. Overall, elevated nitrogen input is detrimental to the buildup of soil organic matter and total nitrogen, and it also influences the content and fluctuations of soil-accessible nitrogen.

The beneficial impact of low nitrogen treatment on bacterial diversity and its association with soil indicators is shown in Figure 5. Encouraging bacterial variety facilitates the development of intricate microbial networks and improves ecosystem functional redundancy [41]; while it adversely affects soil nitrogen, it fosters the buildup of organic matter. Organic matter and alkali-hydrolyzable nitrogen, as critical environmental factors, synergistically influence the microbial community. Organic matter serves as a carbon source and habitat for microorganisms, with its decomposition regulated by nitrogen, influencing microbial growth, metabolism, and community assembly. This creates a synergistic soil–microorganism feedback loop that significantly impacts soil fertility evolution and ecological function maintenance [42]. The combination of exogenous nitrogen fertilizer and the maize rhizosphere effect synergistically influences the nitrogen priming effect and the microbial community. Elevated nitrogen levels suppress bacterial diversity and alter fungal communities. While it enhances soil nitrogen availability, it disrupts the equilibrium of the microbial population, potentially resulting in soil structure degradation, nutrient depletion, and other issues that jeopardize agricultural ecological viability. The nitrogen priming effect alters the microbial habitat and resource availability, thereby precisely regulating the intensity of this effect and the succession of microbial communities, which ultimately influences the stability of soil ecological functions and the efficiency of agricultural production.

Given the extensive prevalence of saline–alkali land globally and the necessity for land resources in agricultural production, the research findings possess significant potential for dissemination and application. In various saline–alkali ecosystems, despite substantial differences in soil composition and climatic circumstances, the fundamental concepts of nitrogen management and microbial regulation are interconnected. In dry saline–alkali regions, the judicious optimization of irrigation, accurate nitrogen administration, inoculation of microbial agents, and rational utilization of nitrogen excitation effects can mitigate salt stress and enhance nitrogen usage efficiency. Based on the attributes of coastal saline–alkali land, salt-tolerant, and submergence-tolerant functional bacteria were selected to enhance soil quality, collaborate with agricultural management to regulate nitrogen activation, and facilitate vegetation regeneration and agricultural advancement. Enhancing nitrogen management and microbial regulatory tactics is achievable in many saline–alkali ecosystems, facilitating the realization of agricultural production potential and augmenting the value of ecosystem services. It holds substantial importance for global saline–alkali ecological restoration and sustainable agricultural advancement.

## 5. Conclusions

This study examined the impact of nitrogen priming effect on the maize rhizosphere microbial population in saline–alkali farmland located in semi-arid regions, leading to the following important conclusions: Nitrogen priming effect considerably altered the organization of the maize rhizosphere microbial population at varying nitrogen levels. The N3 treatment in the bacterial community exhibited significant deviation, and the disparity between the fungal nitrogen application group and the control group was substantial. Elevated nitrogen levels heightened competition and facilitated niche differentiation. The selected characteristic functional genera exhibited notable niche and functional alterations in response to nitrogen priming effect, including *Lysobacter* and *Coniophora*. Their metabolism and soil feedback may serve as critical indicators of soil health. Nitrogen fluctuations influence the intricate relationship between bacteria and microorganisms. Reduced nitrogen levels foster symbiosis, whereas elevated nitrogen levels induce competition. The nitrogen priming effect at varying nitrogen levels influences microorganisms and soil indices differently. Enhancing nutrient management should govern microbial community dynamics and provide precise nitrogen application, which is crucial for the sustained advancement of agriculture in saline–alkali soils.

## Figures and Tables

**Table 1:** Treatments used in this study.

Treatments Coded	Treatments
Control N1 N2 N3	In different fertilization periods, the same disturbance treatment was applied to the soil, but no nitrogen fertilizer was applied. Basal fertilizer: 20 kg N ha^−1^; seventh leaf stage: 40 kg N ha^−1^; tasseling stage: 20 kg N ha^−1^. Total amount of nitrogen fertilizer: 60 kg N ha^−1^. Basal fertilizer: 45 kg N ha^−1^; seventh leaf stage: 90 kg N ha^−1^; tasseling stage: 45 kg N ha^−1^. Total amount of nitrogen fertilizer: 180 kg N ha^−1^. Basal fertilizer: 75 kg N ha^−1^; seventh leaf stage: 150 kg N ha^−1^; tasseling stage: 75 kg N ha^−1^. Total amount of nitrogen fertilizer: 300 kg N ha^−1^.

## Data Availability

Data are contained within the article and Supplementary Materials.

## Supplementary Materials

The following supporting information can be downloaded at https://www.mdpi.com/article/10.3390/plants14121806/s1: Table S1: Basic soil fertility of 0–20 cm plough layer.
