# Supplementary material for: Characteristic Functional Genera (CFG) Mediate Nitrogen Priming Effect in the Microbiome of Saline–Alkaline Farmland
Source: Plants (Basel). 2025 Jun 12;14(12):1806. doi: 10.3390/plants14121806 (PMC12197168; doi:10.3390/plants14121806)
Supplement: Supplementary file 1 [file plants-14-01806-s001.zip › plants-3674910-supplementary.pdf]

Table S1 Basic soil fertility of 0-20 cm plough layer

Parameter	Value
Sand (%)	42.3±3.1%
Silt (%)	35.8±2.5%
Clay (%)	21.9±1.8%
pH	8.17±0.2
Electrical conductivity (EC)	4.2±0.5 dS m^{-1}
Cation exchange capacity (CEC)	12.5±1.3 $cmol^{+}$ kg^{-1}
Organic matter (OM)	25.14±1.8 g kg^{-1}
Total nitrogen (TN)	1.221.2±0.1 g kg^{-1}
Available phosphorus (AP)	103.76±0.6 mg kg^{-1}
Available phosphorus (AP)	16.23±0.7 mg kg^{-1}
Available potassium (AK)	106.16±10 mg kg^{-1}
Water holding capacity (WHC)	35.4±2.1%
Bulk density	1.4±0.1 g cm^{-3}
